# Pesticide–Virus Interactions in Honey Bees: Challenges and Opportunities for Understanding Drivers of Bee Declines

**DOI:** 10.3390/v12050566

**Published:** 2020-05-21

**Authors:** Gyan P. Harwood, Adam G. Dolezal

**Affiliations:** Department of Entomology, University of Illinois at Urbana-Champaign, Urbana, IL 61801, USA; adolezal@illinois.edu

**Keywords:** honey bee virus, virus tolerance/resistance, pesticide resistance, toxicology, insecticide–virus synergism

## Abstract

Honey bees are key agricultural pollinators, but beekeepers continually suffer high annual colony losses owing to a number of environmental stressors, including inadequate nutrition, pressures from parasites and pathogens, and exposure to a wide variety of pesticides. In this review, we examine how two such stressors, pesticides and viruses, may interact in additive or synergistic ways to affect honey bee health. Despite what appears to be a straightforward comparison, there is a dearth of studies examining this issue likely owing to the complexity of such interactions. Such complexities include the wide array of pesticide chemical classes with different modes of actions, the coupling of many bee viruses with ectoparasitic *Varroa* mites, and the intricate social structure of honey bee colonies. Together, these issues pose a challenge to researchers examining the effects pesticide-virus interactions at both the individual and colony level.

## 1. Introduction

Honey bees (*Apis mellifera*) are perhaps the most important insect for human well-being, helping to pollinate over $200 billion USD in agricultural crops per year [1]. Yet, despite their importance to agriculture, honey bees continue to suffer substantial annual colony losses in many countries, with American beekeepers alone losing more than 50% of their colonies in 2018 [2]. Despite considerable effort, no single ‘smoking gun’ for these losses has appeared; instead, high losses have been linked to several co-occurring biotic and abiotic environmental stressors. These include poor nutrition due to habitat loss and low floral quantity or quality in agriculture systems, parasite pressures from *Varroa destructor* mites, immune challenges from a suite of viruses and other pathogens, and exposure to numerous pesticides [3,4,5,6,7,8]. Adding further complexity to the issue, many of these stressors act simultaneously on honey bees and can exert additive or even synergistic effects [9,10,11,12,13,14,15,16]. For example, dietary pollen quality and quantity greatly affects immunocompetence, and bees with poor nutrition are more susceptible to parasites and pathogens [17,18,19].

In this review, we focus on the interaction between two stressors that has thus far received surprisingly little attention: that of pesticides and viruses. We first briefly discuss the broad range of chemical classes used by farmers, public health officials, and beekeepers to control pest populations, the modes of action by which these chemicals target insects, and the ways bees can be affected by sublethal doses. We then review our current knowledge of bee viruses, the immunological pathways used by bees to fight infection, and the ways viruses are transmitted between individuals, colonies, and even species. Finally, we examine how some pesticides do (or do not) promote viral replication or pathological effects at both the individual and colony level, and highlight areas of future research needed to fill knowledge gaps.

## 2. Pesticides

“Pesticide” is a broad term denoting any substance that is used to eliminate pest species and can include insecticides, herbicides, fungicides, and nematicides. Pesticides represent a diverse array of chemical classes with different modes of action, and as such, examining the effects of pesticides on honey bees is not a straightforward endeavor. Adding further complication, honey bees often encounter many different chemicals simultaneously [20,21,22,23] owing to their ubiquity in commercial pollination, their generalist foraging strategy, and their large foraging ranges that can cover hundreds of square kilometers [24]. These different chemicals, along with adjuvants and other additives in the applied formulations, can interact with one another to produce additive or sometimes synergistic effects in bees and other insects [12,25,26]. Much work has been done examining the acute toxicity and lethal dosages of these pesticides, as such measures are required by regulatory agencies for product registration [27], but bees often encounter pesticides at sublethal doses in their environment. Even these lower doses can produce various effects in bees, including impairments to behavior [28,29,30,31], learning and memory [32,33,34], longevity [35], and immune function [36]. Here, we briefly outline some of these chemical classes commonly encountered by bees, the sublethal effects they exert on bees, as well as the modes of actions of these chemicals in bees or other more common insect models, such as fruit flies and mosquitos.

### 2.1. General Background on Classes of Pesticides

Many commercial insecticides are synthetic analogs of naturally-occurring chemical compounds produced by plants and often act by disrupting the nervous system or muscle tissue function [37,38]. While a full discussion of all these compounds is beyond the scope of this review, comprehensive reviews can be found elsewhere [39,40]. Organophosphates and carbamates are widely used in agriculture and pest prevention and disrupt nerve function by inactivating acetylcholinesterase, an enzyme used to clear acetylcholine neurotransmitters from the synapse [40]. Both classes of chemicals have a broad range of toxicity towards honey bees [41], but one of the most commonly used in crop protection, chlorpyrifos, is highly toxic to bees [42] and often found in hive materials [43]. Even at doses far below the LD_50_ (i.e., the dosage that kills half of the subjects), chlorpyrifos has negative impacts on bees’ appetitive olfactory learning and memory [43]. Likewise, the organophosphate naled is mainly used to control mosquito populations, and incidental exposure in honey bees can lead to increased mortality and lower honey production [44]. Organophosphates and carbamates have been linked to many bee poisoning incidents in the UK [45].

Pyrethroids are another popular class of insecticides and are similar to the natural pyrethrin compounds produced in chrysanthemum plants. They target the insect nervous system by delaying the closure of voltage-gated sodium channels [40], leading to the loss of motor function, paralysis, and ultimately death (reviewed in [46]). They also display a broad range of toxic effects to honey bees [41,47]. Among the most common encountered by bees are bifenthrin and lambda-cyhalothrin. Bifenthrin is used in orchard agriculture and other sectors and at sublethal doses can impair larval development and queen fecundity [48]. Lambda-cyhalothrin, meanwhile, is used to protect a variety of crops and at sublethal doses has been shown to impair honey bee worker longevity, homing ability, and learning and memory [49].

The class of insecticides garnering most public and research attention in the past decade is the neonicotinoids, which are based on analogs to a natural plant-produced alkaloid, nicotine. These compounds act as acetylcholine receptor agonists to disrupt nerve function, and can be taken up systemically by crop plants via seed coatings and irrigation or applied as a foliar spray [40]. Among the most common neonicotinoids that bees encounter are imidacloprid, clothianidin, and thiamethoxam. There has been much concern about the negative effects these neonicotinoids may have on honey bees, but the evidence is somewhat mixed. While some studies have shown that neonicotinoid exposure can compromise foraging activity and survival in honey bees [30,50,51], the concentration of neonicotinoids in the nectar of treated plants may not be high enough to elicit such effects [52]. However, other studies have shown that bees housed near corn fields treated with neonicotinoids show reduced longevity and hygienic behavior [53].

In contrast to neurotoxic compounds that target adult insects, some insecticides target developing larvae by disrupting their molting and cuticle formation processes. These insect growth regulators (IGRs) are typically synthetic analogs of juvenile hormone or ecdysone, two hormones critical for development and molting, or they are chitin inhibitors that interfere with the formation of the exoskeleton. Thus, the production of viable new offspring can be greatly impeded. IGRs may have little to no lethal effects on adult honey bee workers [54], but they can disrupt their behavioral development [55,56] and have been shown to affect egg viability and larval development [56,57]. For a review of IGRs see [58].

Honey bees also encounter non-insect-targeting pesticides, such as herbicides and fungicides, which were long presumed to have little to no effect on bees. However, more recent studies suggest they pose some risk. Herbicides can indirectly impact honey bee health by limiting the availability of diverse floral sources [59], thus compromising the bees’ nutritional state, which can lead to further compromises to immune system function [60]. However, herbicides and fungicides can also exert more direct effects. For example, glyphosate is widely used to eliminate weeds in corn and soy production, but it can disrupt honey bee gut microbiota that play important roles in immunity and weight gain [61], as well as affect larval development [62] and worker behavior [63]. Interestingly, honey bee foragers show a preference for sucrose solution with low concentrations of glyphosate over control solution [64]. Atrazine, another popular herbicide that is frequently found in hive material [65], has been shown to reduce levels of some antioxidants in honey bees [66]. Azoles, a common class of fungicides, have been shown to affect ATP production in honey bee flight muscle [67], which in turn can impede bees’ abilities to thermoregulate [68]. Moreover, fungicides are often applied concurrently with insecticides in agriculture, and co-exposure experiments show synergistic effects on lethality and increased risk to honey bees [69].

It is also important to consider, however, that the findings discussed thus far represent just a snapshot of documented effects of specific pesticides on honey bees. Other effects may remain undiscovered because scientists have not yet looked for them. For example, neonicotinoids’ effects on honey bee health have been studied intensively, but older classes of pesticides like pyrethroids and organophosphates have received less attention. New information may also prompt re-examination of previously studied pesticides, as is the case with fungicides, which were considered harmless to bees but which are now understood to act synergistically with certain insecticides. Furthermore, some pesticides may elicit no effects in a particular study [70], but such non-effects may be less documented due to publication bias [71]. Undiscovered and non-effects could be important for understanding interactions between pesticides and the immune system, but scientists will have to overcome this information barrier.

### 2.2. How Bees Detoxify Pesticides

A pesticide’s toxicity and sublethal effects depend on how an organism metabolizes and detoxifies the particular compound. There are several families of enzymes involved in detoxification pathways, but the most prominent and well-studied are the cytochrome P450 monooxygenases [72]. Detoxification is typically initiated when a P450 enzyme targets specific chemical structures on the xenobiotic molecule and catalyzes its reduction into metabolites. These metabolites are then bound by other enzymes that allow for transport and eventual secretion of the metabolite. Some pesticides can interfere with the P450 function [73], and some synergists are specifically added to insecticide formulations for this purpose to increase toxicity towards target species [74]. A given pesticide’s toxicity is also not consistent among all honey bee colony members, with larvae being especially susceptible [48,62,75,76,77,78,79]. In fact, pesticide toxicity can vary with many factors, including caste [80], age [81], season [82,83], genetics [81], and nutritional state [83]. In this latter regard, plant pollens and nectars contain many phytochemicals that up-regulate the expression of detoxification genes and can increase bees’ tolerance to some pesticides [84,85]. It should also be noted that honey bees have fewer detoxification genes than solitary insect species [86,87].

### 2.3. Active Ingredients vs. Formulations

**Figure 1:**
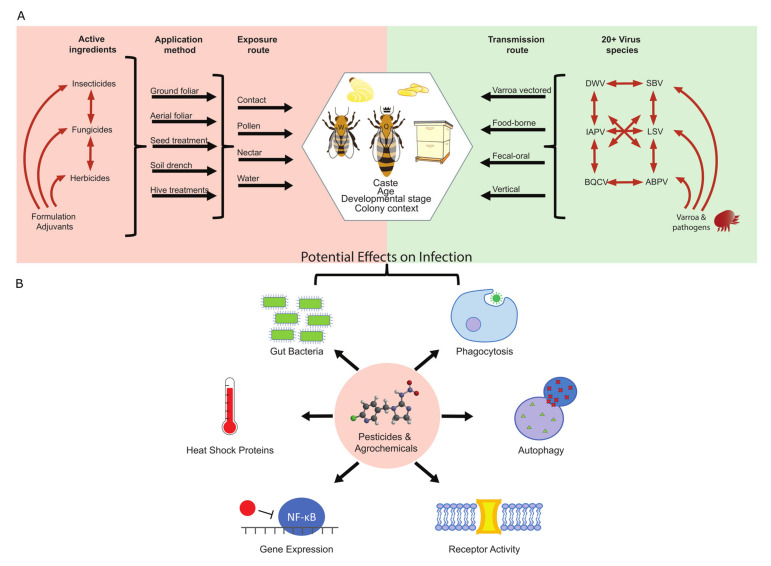
(**A**) Pesticide × virus interactions require integration of many potential scenarios, and there are many challenges in understanding the relevance of potential interactions. Pesticide considerations: Honey bees can and have been exposed to many different pesticide types (insecticides, fungicides, herbicides, etc.), each containing multiple classes with different modes of action. Product formulations also contain adjuvants and “inert” chemicals that can interact with active ingredients to alter pesticide sensitivity in bees. One must also consider how application methods might affect how bees are exposed to the pesticides. Virus considerations: honey bees can be infected with more than 20 different types of viruses, often at the same time, and there are likely coinfection interactions (DWV: deformed wing virus; SBV: sacbrood virus; IAPV: Israeli acute paralysis virus; LSV: Lake Sinai virus; BQCV: black queen cell virus; ABPV: acute bee paralysis virus). At the same time, variable pressure from *Varroa* mites and other pathogens can affect these dynamics. The route of exposure to these viruses is likely to cause different responses in the infected bees. Overall considerations: for both pesticide and virus experiments, the context of investigation can also yield variable responses. Bees of different caste (queen vs. worker), developmental stage (egg, larva, pupa, and adult), and age can all experience these stressors differently. Further, most experimental manipulations of pesticide and virus stress are done outside of the colony context; honey bee colony units can respond differently than individuals or small groups in experimental settings. Thus, studying pesticide/virus interactions in honey bees sounds deceptively simple; however, the potential interactions make investigation a significant challenge. (**B**) Pesticide exposure can negatively impact many components and pathways of the immune system. Phagocytosis: pro-hemocyte differentiation can be impaired, resulting in fewer phagocytosing immune cells. The process of phagocytosis itself can also be affected; autophagy: Regulation of autophagy can be disrupted, potentially leading to apoptosis in cells; receptor activity: some insecticides target receptors that are also involved in antiviral defenses; gene expression: pesticides can alter expression of immune and detoxification genes. This includes upregulating inhibitors of the important immune system transcription factor, NF-κB; heat shock proteins: some pesticides downregulate expression of genes coding for heat shock proteins. These proteins can reduce viral load and also have functions in the RNAi antiviral pathway; gut bacteria: pesticides can also disrupt gut microbial communities, which are known to play roles in honey bee health and immunity.

Pesticide toxicology studies in honey bees can be complicated by how the commercial products are formulated. While many experiments use pure active ingredients, real world applications of formulated product also contain adjuvants with potential effects on bees. These all have the potential to interact with each other, and even ingredients considered “inert” can negatively affect bees [26,75,88]. Identical active ingredients can also have different formulations for different applications. Each of these has its own potential risks and can all contribute to different potential routes of exposure to the bees—through direct drift of chemicals onto bees/hives, contamination of water, and contamination of diet via direct exposure or systemic plant movement (Figure 1A).

### 2.4. Treating for Varroa mites

Honey bee exposure to pesticides is often incidental, being a consequence of farmers, public health officials, and even homeowners seeking to eliminate pests. However, beekeepers also intentionally apply some chemicals to treat for pests within bee colonies. These include treatments for small hive beetle (*Aethina tumida*), wax moths (*Galleria mellonella* and *Achroia grisella*), and tracheal mites (*Acarapis woodi*), but are mostly focused on control of *Varroa* mites. Several miticides have been used historically, including coumaphos (an organophosphate), τ-fluvalinate (a pyrethroid), amitraz (an adrenergic agonist), and several organic acids and essential oils (e.g., Thymol), though some of these, like coumaphos, have become uncommon due to resistance evolution [89]. Despite clear benefits in controlling parasites, each of these chemicals does pose some degree of risk for honey bees. For example, coumaphos exposure can lead to increased larval mortality [75,90,91], τ-fluvalinate exposure can affect learning and memory [92] and larval and adult survival [75,91], and thymol exposure can lead to brood removal by workers [93] and decreased larval survival at high doses [94]. Despite these risks, these treatments are still viewed as critical, as *Varroa* pose an outsized risk to honey bees and are a primary cause of annual colony losses [95,96]. *Varroa* mites have negative impacts on many important aspects of honey bee biology, including physiology [97], behavior [98], and nutritional state [99,100] (see [101,102] for comprehensive reviews). However, perhaps their biggest threat to honey bees come from their role as vectors for some of the most prolific and deadly honey bee viruses.

## 3. Honey Bee Viruses

Dozens of honey bee viruses have been characterized, with the vast majority being positive-sense single-stranded RNA viruses (+ssRNA). More exhaustive reviews than the one provided here can be found elsewhere, e.g., [103]. Among the most prevalent viruses, many belong to the *Dicistroviridae* family, including Israeli acute paralysis virus (IAPV), acute bee paralysis virus (ABPV), Kashmir bee virus (KBV), and black queen cell virus (BQCV) [104]. IAPV, ABPV, and KBV are very closely related and often considered as a virus complex [105]. Many other bee viruses belong to the *Iflaviridae* family, including deformed wing virus (DWV), sacbrood virus (SBV), and slow bee paralysis virus (SBPV). *Varroa* mites are vectors for many of these viruses, and two in particular, IAPV and DWV, have been associated with high colony losses [7]. IAPV symptoms include quivering, paralysis, and eventual death, and contracting the virus can be lethal to bees of all ages and developmental stages [106,107]. DWV infection during pupal development can lead to emerging adult workers with under-developed and non-functional wings, altered behavior, and decreased lifespan [108], which contribute to high winter colony losses. However, workers that contract the virus as adults do not appear to show overt symptoms [108]. DWV virulence also appears to be amplified by *Varroa* mites. Like most RNA viruses, there are many strains of DWV owing to high sequence variation (so called quasispecies) [108], and transmission via *Varroa* may be selecting for more virulent strains. Laboratory studies show that even when mites have a high diversity of replicating DWV strains, the infected pupae show increased replication for a single virulent strain [109,110]. This has also been observed in the field, where the high diversity of DWV strains in Hawaiian apiaries was greatly reduced when *Varroa* mites were introduced to the islands [111].

### 3.1. Sublethal Infection

Viral infections are extremely prevalent in honey bee colonies, with recent surveys showing DWV infection in 89% of US colonies [112] and 100% of English/Welsh apiaries [113]. Most bees harbor viral infections at subclinical levels, meaning the viral loads are such that individuals do not exhibit any overt symptoms. In many cases these low-level infections do not appear to negatively impact a colony’s growth rate, but adequately studying the impacts of asymptomatic infection is challenging due to the scarcity of proper non-infected control colonies. Typically, an individual bee’s immune system is able to control viral replication, but when particular environmental stressors increase in severity, they can compromise a bee’s immune system and release viral replication. For example, bees without access to diverse floral resources may lack important proteins and lipids, potentially allowing viral replication to increase [16]. Lab-reared adult workers that feed exclusively on sucrose end up with higher viral titers than workers whose diets are also supplemented with protein-rich pollen [16]. Sublethal infections also cause changes in gene expression patterns, some of which are due to the host’s antiviral response [114], but some may be due to virus manipulation of the host to promote transmission between colonies [115] (see Section 3.2).

### 3.2. Viral Transmission

Within a honey bee colony, viruses can be transmitted in a multitude of ways and seemingly exploit all routes of material exchange between colony members. Horizontal transmission between nestmates can occur when workers exchange saliva via food sharing (trophallaxis) and when they feed the queen and young larvae with a glandular secretion called royal jelly. Both saliva and royal jelly test positive for viral copies [114,116,117]. Horizontal transmission can also occur via sexual contact between drones (males) and the queen [114,118], and by physical contact between nestmates [117]. Vertical transmission is also present, as evidenced by viral copies in the queens ovaries and eggs [114,118]. However, *Varroa* mites remain the most problematic and well-documented route of bee viral transmission. *Varroa* can parasitize bees of all castes and developmental stages (excluding eggs and L1–L4) but given their nature as obligate parasites they primarily feed on the pupal stage when larvae are capped inside brood cells during their metamorphosis. Here, female mites lay eggs that will hatch before the end of pupation and the resulting offspring can spread to other larvae or adult bees within the hive, thereby exhibiting exponential population growth. *Varroa* mites also facilitate inter-colony viral transmission by attaching themselves to workers and drones that exit the hive and potentially switching hosts at floral forage patches. Further still, *Varroa*-vectored viruses can actually move between species [119,120], threatening vulnerable native bees [121]. Viruses can also transfer between hives without a *Varroa* vector, and viral infection may actually alter host behavior and chemical signature to help facilitate this process [115]. Within a hive, IAPV-infected workers receive fewer social interactions from their nestmates, but they are more likely than non-infected individuals to be permitted into foreign hives. Here, infected workers that leave the hive to forage are more likely than non-infected workers to return to a foreign hive, a process known as drifting. Drifting occurs under normal circumstances in densely packed apiaries, and drifters are typically turned away from foreign hives by a team of guard bees that monitor the hive entrance. However, infected bees are permitted into foreign hives at higher rates than is typical, possibly due to changes in their chemical signature encoded in their cuticle [115].

### 3.3. Antiviral Immune Pathways

Honey bees, like all insects, have several highly conserved innate immunological pathways to fight pathogens. This includes the RNA-interference (RNAi) pathway [122], which evolved specifically to combat viruses [123]. Here, the enzyme Dicer targets double-stranded RNA (dsRNA), as this type of molecule is typically only found in eukaryotic cells during viral replication. Dicer cleaves the dsRNA into smaller RNAs, after which these small RNAs are unwound and used as a template to find specific complementary sequences of single-stranded RNA (e.g., mRNA). These mRNAs are enzymatically cleaved, ultimately reducing viral replication and expression of viral genes. Researchers can exploit this RNAi pathway to knockdown gene expression by administering dsRNA to organisms, and feeding dsRNA to bees (both virus sequence-specific or non-specific alike) has been shown to reduce viral loads of bee pathogens like IAPV [124,125]

Insects also possess other conserved innate immune system pathways, including the immune deficiency (IMD), Toll, and Jak-STAT (Janus kinase and signal transducer and activator of transcription) pathways. These pathways have well-characterized responses against cellular pathogens like bacteria and fungi, but are also activated during viral infection [126,127]. Here, pathogen-associated molecular patterns (PAMPs) are first bound to pathogen-recognizing receptors, which initiate a cascade of reactions that often induces expression of antimicrobial peptides (AMPs). Both the Toll and IMD pathways share an important component, the transcription factor NF-κB (nuclear factor kappa-B), which translocates from the cytosol to the nucleus to upregulate AMP expression [128]. Prominent model systems like *Drosophila melanogaster* and various mosquito species have been used to show the IMD and Toll pathways playing direct antiviral roles [129,130,131,132]. For example, *D. melanogaster* injected with cricket paralysis virus (CrPV) experience depleted reserves of hemocytes (immune cells) over the course of infection, while IMD loss-of-function mutants are more sensitive to infection and display higher viral loads [129]. Similarly, Toll loss-of-function *D. melanogaster* mutants display higher viral loads and increased susceptibility compared to wildtype individuals when exposed to viruses [131].

Other immune system components involved in antiviral defense include autophagy, phagocytosis, and heat shock proteins [133,134,135]. Autophagy is an intracellular mechanism for degrading and recycling cytoplasmic components and occurs through the formation of double-membraned vesicles. These vesicles then fuse with lysosomes and their internal contents are degraded. This process is essential for fighting some insect viruses [133]. Phagocytosis is a cellular process by which certain hemocytes (i.e., macrophages/plasmatocytes) degrade dead, infected, or pathogenic cells, and this response can be crucial in eliminating virus-infected cells [134]. Heat shock proteins are part of the organismal stress responses, but their genes are also induced during viral infection to help reduce replication [135]. They also act as chaperones to help facilitate the RNAi antiviral pathway [136]. For more comprehensive reviews of insect antiviral pathways, see [126,127].

In addition to individual-level immune defenses, honey bees also employ colony-level defenses through a suite of behavioral and physiological traits termed social immunity. Individuals can prevent or slow the spread of pathogens by isolating or removing infected individuals, grooming each other for parasites, sterilizing the nest and food by distributing antimicrobial plant resins and secreting glucose oxidase enzymes throughout the hive material and food, and many other phenomena [137,138]. Social immunity can also protect future offspring through a process called trans-generational immune priming (TGIP) [139]. Here, a female that survives a pathogen attack can transfer PAMP-containing particles to her eggs [140], which primes the developing immune system to produce more resistant offspring. In honey bees, recent findings suggest the sterile worker caste may participate in TGIP by incorporating PAMP-containing particles into the royal jelly glandular secretions they feed to the queen and young larvae [141,142].

## 4. Interaction of Pesticides and Honey Bee Viruses

While honey bees and other insects have evolved mechanisms to detoxify xenobiotics like pesticides and fight pathogen threats like viruses, real-world conditions mean that honey bees are often exposed to these environmental stressors simultaneously. The question then becomes, does pesticide exposure lead to increased viral transmission or virulence? Perhaps unsurprising, given the wide variety of pesticides and pathogens encountered by bees in different contexts, investigations have provided mixed results, with many studies showing pesticides exerting additive or synergistic effects on virus-induced mortality and replication in honey bees [36,143,144,145,146,147], and others showing little to no effect of pesticide exposure on viral infections in honey bees and bumble bees [148,149,150,151].

### 4.1. How Pesticides Can Impact Antiviral Pathways

To understand why pesticides may increase susceptibility to viruses, it is important to know how they affect antiviral immune pathways (Figure 1B). Again, much of this work has been performed in other prominent insect models like *D. melanogaster* and various mosquito species. First, pesticide exposure can disrupt cellular immune defenses by affecting hemocyte differentiation and function. Larval *D. melanogaster* fed with acephate (an organophosphate) display altered differential hemocyte counts, with a relative decrease in phagocytosing macrophages/plasmatocytes [152]. Moreover, exposure to the neonicotinoid imidacloprid can reduce phagocytotic function of hemocytes [153,154], and also decrease the survival of fat body cells, which play critical roles in immune responses such as AMP production [155]. Some pesticides have also been shown to disrupt important intracellular pathways involved in immunity, like autophagy [156].

Second, numerous studies have highlighted the impact pesticides have on expression of detoxification and immune genes [155,157]. This includes downregulating gene expression of immunologically-relevant heat shock proteins [158]. One can additionally learn how pesticides affect immune function by comparing pesticide-resistant and wild-type strains of insects. Permethrin-resistant mosquitos upregulate expression of several detoxification and immune genes after exposure to the pesticide compared with wildtype mosquitos [159], and resistant mosquitos likewise upregulate detoxification and immune genes after Zika virus infection while wild-type mosquitos downregulate many of these genes after infection [160]. Effects on gene expression and subsequent protein levels may be pesticide- and tissue-specific. For example, mosquitos exposed to temephos (an organophosphate) show upregulation of dozens of proteins in the midgut, including many immune proteins [161].

Third, due to crosstalk and interconnectedness between various immune pathways [162], pesticides that affect gene expression in one pathway may elicit downstream effects in other pathways. For example, exposure to imidacloprid in *D. melanogaster* can down-regulate components of the IMD pathway that help to regulate the DUOX pathway [163,164]. The DUOX pathway produces hydrogen peroxide, which is used not only to kill pathogens but also to regulate gut microbiota composition [164], and disruption of this microbiome can increase susceptibility to some pathogens [165]. In honey bees, the herbicide glyphosate [61,166] is known to disrupt gut microbial communities, although exposure to imidacloprid may not produce such a change [167].

Finally, some pesticides can directly increase viral susceptibility in insects by targeting ion channels that play a role in antiviral defense. In an effort to combat pesticide-resistance in mosquito vectors of human diseases, promising new research has targeted inward rectifier potassium channels [168]. However, these channels have been shown to regulate infection of cardiotropic viruses in *D. melanogaster* [169], and honey bees show similar increases in mortality and viral replication when these receptors are blocked [170]. The development of novel insecticides is important for overcoming resistance but it poses a challenge to scientists who must once again discover all the off-target effects of a new chemical class.

So, does exposure to pesticides increase honey bees’ susceptibility to viruses? As previously mentioned, results are mixed and may depend on the specific pesticides and viruses being tested, the manner in which bees are exposed to pesticides, and whether researchers are focused on individual- or colony-level effects. To date, most studies examining pesticide-virus interactions in honey bees have focused on neonicotinoids, likely owing to the public concern that this class of chemicals may pose risks to pollinators. In a landmark study, Di Prisco et al. [36] showed that viral replication of DWV was increased in individual bees exposed to the neonicotinoids clothianidin and imidacloprid, but not in bees exposed to the organophosphate chlorpyrifos. Furthermore, clothianidin exposure upregulated an inhibitor of the important immunological transcription factor NF-κB, thereby providing a mechanistic explanation for how some pesticides can weaken host antiviral defenses and impart synergistic effects on viruses [36]. In subsequent studies, honey bees exposed to neonicotinoids like imidacloprid, clothianidin, and thiacloprid showed reduced hemocyte counts, reduced encapsulation response, and reduced antimicrobial activity [171], providing further insight into possible mechanisms of reduced antiviral defenses.

In another study looking at BQCV infection and thiacloprid exposure, researchers found additive effects of virus and pesticide in honey bee larvae when orally inoculated with high doses of the virus, but found no such effect in adult workers [146]. Several studies have also looked at the possible interactions between CBPV and various neonicotinoid pesticides, with varying results. Coulon et al. [143] orally administered thiamethoxam to summer-born worker bees and housed them with overtly infected bees, observing that high pesticide doses had a synergistic effect on virus-induced mortality without an increase in viral titers, while low pesticide doses exerted no synergistic effects on mortality but did cause an increase in viral titers. A follow-up study in winter-born bees showed no synergistic effect on mortality, but they did observe an increase in viral titers to a level typically seen in overt infections [144]. Similarly, Diao et al. [145] fed varying doses of imidacloprid to CBPV-inoculated spring bees and found that high pesticide doses elicited synergistic effects on viral titers and mortality, but that low pesticide doses had no discernable effect on these virus parameters. Other classes of pesticides remain less studied in terms of their effects on honey bee immunology. IGRs have been shown to have additive lethal effects with some insect pathogens [172,173,174], but to our knowledge there has been no demonstration of synergistic effects. Besides insecticides, other studies have observed synergistic effects on virus-induced mortality when honey bees are co-exposed to a fungicide [147] or a common adjuvant added to many pesticide products [88].

### 4.2. Laboratory- vs. Field-Based Studies

Despite evidence from laboratory experiments that pesticides can affect virus infections in bees, these findings do not always corroborate results from field-based studies that track changes in natural virus titers. Several large-scale studies have looked at honey bee hives placed near fields of clothianidin-treated canola (rape-seed) and found no increase in titers of several viruses [148,149], nor an increase in expression of immune genes [149], nor an increase in prevalence of overt viral symptoms [175]. A similar study in bumble bees also showed no increase in viral titers when hives were placed near clothianidin-treated fields [151]. In addition, some studies have shown that hives supplemented with imidacloprid in their diet did not experience significant population losses compared to control hives [176], and others found no correlation between colony health and viral prevalence at agricultural sites [150].

This apparent discrepancy between laboratory- and field-based pesticide exposure assays illustrates how, at the colony level, honey bees can be very resilient against environmental stressors and their responses to pesticides can be context-dependent. For example, imidacloprid exposure to laboratory-reared bees can down-regulate expression of immune and detoxification genes while hive-reared bees show up-regulation in several of these genes [177]. This discrepancy represents a major challenge for researchers trying to understand how bees respond to environmental stressors, and there are several factors that likely contribute to these differences in response. Firstly, honey bees live in highly structured societies, with a reproductive queen and a worker caste that progresses through age-related tasks in response to physiological, chemical, and social cues. While pesticide exposure can decrease lifespan of individual bees, a colony-level response can shift the demographics within a hive and mask many impacts [178]. Likewise, laboratory settings can better control extraneous variables, but a disruption to important social and chemical cues likely adds significant stress on the bees and may suppress their immunocompetence. Second, honey bees have a reduced number of immune and detoxification genes compared with solitary species [86,87], but, as already discussed, they also exhibit social immunity. Such social immunity mechanisms are likely lacking or absent in lab-reared bees that comprise a single age cohort. Finally, lab-reared bees are typically fed a restricted diet of sucrose solution, as opposed to the myriad nectars and pollens collected by hive-reared bees, and as already discussed a restricted diet can negatively impact immunity [16].

While field-based studies more accurately capture the day-to-day conditions experienced by bees, there are significant challenges in developing adequate experimental designs, especially in terms of sample size and proper controls. It is far easier to generate large samples of individual bees in a lab than it is to have a sufficient number of hives at various field sites. Two recent studies looking at the effects of field-realistic exposure to neonicotinoids on honey bee health illustrate the magnitude of experiments required to conduct adequate studies [53,179]. Trying to control variables like pesticide exposure is difficult, given that honey bees have large ranges and are generalist foragers. Hives placed in or near pesticide-treated fields will likely still gather materials from non-treated areas, and likewise, hives in or near untreated fields can still forage in treated areas and also be exposed to other pesticides applied by private citizens or public health officials. Treated and untreated sites can also differ in the types and diversity of plants available, adding further inconsistencies between sites. Adequate experiments require high site-type replication to overcome issues with such confounding variables, which places strains on research teams to manage such a large number of hives.

## 5. Future Directions

Despite the many challenges of studying pesticide-virus interactions in honey bees (Figure 1), continuing this research is critical for maintaining food security, particularly as agroecosystems face pressures from climate change. There are several aspects of this issue for which our knowledge must improve. First, we need a greater understanding of how innate immune system pathways such as Toll and IMD operate on a molecular level to fight viruses. We know that these pathways are activated upon viral infection and that knocking out certain components increases virus replication and lethality, but we still lack some mechanistic explanations for how the viruses are destroyed or prevented from replicating. Knowing these mechanisms may help researchers design treatments for current honey bee pathogens and novel pathogens that may arise in the future. Second, we must continue to identify immune and detoxification pathway components that are directly affected by pesticides in order to predict how exposure will affect host antiviral responses. Here, researchers could make uses of advances in machine learning and metabolic modeling to aid their search. Finally, we must carry out pesticide exposure and viral inoculation assays in both laboratory and field settings, as effects observed on individuals in the lab may be buffered by the group-level responses of whole colonies. In a perfect world, one would study a matrix of effects, examining a slew of viruses and pesticides administered in various combinations, doses, and exposure methods, but such endeavors are daunting. Perhaps instead, researchers can first turn to the plethora of immunological and detoxification studies performed in prominent insect models like *D. melanogaster*, mosquitos, and major crop pests. Here, an understanding of how these pathways are affected by pathogens and xenobiotics in one model organism can inform researchers’ hypotheses of how they may be affected in honey bees. Such hypotheses can first be tested in laboratory settings, and if effects are observed then the study can be expanded to determine if such effects persist at the colony level in the field. These types of studies will be particularly important going forward as new classes of “pollinator-friendly” pesticides are developed. Even those deemed relatively safe for honey bees may exert sublethal effects that impact honey bee health [25,180]. Challenges will always remain when studying a highly social species in landscapes filled with multiple co-occurring stressors, but honey bees’ great importance to human well-being necessitates that such efforts continue.
